# The Physician as Pessimist

**Published:** 1898-10-01

**Authors:** 


### Editor's Letter-Box.

[Our Correspondents are reminded that prolixity is a great bar to publication, and that brevity of style and conciseness of statement greatly facilitate early insertion.]

### THE PHYSICIAN AS PESSIMIST.

G. A. Hawkins-Ambler, F.R.C.S.E., Hon. Surgeon to the Liverpool Samaritan Hospital for Women, writes: I recently met in consultation a very intelligent and thoughtful country practitioner, with whom, while waiting for my train, I enjoyed a very interesting conversation. One of the topics that cropped up was the question of the advantages and disadvantages of consultations, and he rather startled me by the remark that he rarely learnt anything from the physician who, since his day at college, some fifteen years before, had, apparently, nothing to offer that was really new and helpful in treatment to the village practitioner who was outside the swirl and vortex of city and hospital practice. Had the statement been made by anyone else, by one less favourably known to me, I should have put it down to the conceit of ignorance that, founding "experience" on no scientific basis, is content to flatter its own stupidity with the hope of successfully coping with emergencies that, if they ever arise, are either ignored or for various casuistic reasons handed over to the nearest consultant. But my friend was not of this class. He follows medicine and surgery with a steady and intelligent interest, and his remark deserves consideration.

Not even a physician would pretend that medicine has made anything like the advance in treatment secured by surgery in the last fifty years. But I question if many physicians would own that, so far as treatment is concerned, there has been any very real advance, or that from the glittering haze enveloping the new country which medicine is now exploring, anything great is likely to crystallise out. We may be like the traveller on the Righi, who, lost in the mist, sat hopelessly with his back against the hotel wall till the fog lifted. But in the meantime, so far as I can follow the practice and experience of my friends, who follow pure medicine, there seems to be, rather than a hopeful spirit abroad, a profound and demoralising pessimism.

It must be demoralising to the student who passes round the wards of his hospital, listens to long and brilliant discourses on pathology and symptoms, and sees the case left with either a placebo or a sneer at treatment of any kind. I am far from saying that this medical agnosticism is as bad as the fatuous playing with so-called "treatment" of the genial imbecile whose "treatment" consists in harping on a new fad of ætiology every three months, and fitting his cases into it for treatment till the new craze comes up. At one time he will babble of gout, at another he is mad on hysteria; again, he seizes a half-truth in physiology or pathology, and fills up his patients with weird animal extracts; anon, he relieves subsidiary symptoms with an intestinal antiseptic, and thenceforward his post-mortem tables reek of pretty new drugs he has poisoned his clients with. The pessimist, at least, leaves his unhappy subject alone while he is following the much more fascinating pursuit of hunting the snar—I mean bacillus—in his laboratory, and putting salt on its tail; but the treatment faddist is a terror to all but the old lady who cannot get enough medicine—the medicine tippler.

But surely, between this outrage on scientific medicine and the pessimism of the usual laboratory physician, there is some working theory of medicine which, without quackery, will put hope into the patient, and, still better, in his doctor.

It is painful to see the blighted air of our young physicians. I cannot believe it is all due to the cheap consultations of their seniors which for years keep them at starvation point. They are not like the young surgeon, who cannot afford to take the absurdly small fees that old surgical hands sell their reputations for, and who has to keep up the reputation of the profession on the pittance that the rare substantial fee spreads out into. I rather fancy that want of success, while not without its importance, has not all to do with this appearance of despondency and despair, and that the wan, wild smile of the follower of pure medicine, who is not more hard up than his surgical confrère, is due to his practising a branch of medicine that is as hopeful for him as the search for the philosopher's stone. The old physician has found this out. He encourages research and the researcher with a very occasional case and a rare fee for microscopical investigation. While he studies humanity, the humanity that holds the germs, and pockets the ready fee, his young friend is making cultures of the unremunerative bacillus and forgetting that such a thing as a human being is in existence. But is it not the human being who pays as a study, and, in medicine as in life, the proper study of mankind is man. The specialised, exclusive devotion to pathology as a study, coupled with the necessarily neglected observation of the man, the woman, the child, and the idiosyncrasies of temperament that influence health, means the despair of the patient and crippling of the resources of the physician.

Does not the quack "cure" his patients, not only very often, but perhaps as often as the physician whose failures he advertises? It is a hard saying, but is capable of considerable demonstration, that the cheery optimism of ignorance or rascality has a curatively bracing effect on the invalid who has grown despondent under the indifference or pessimism of physicians, who probably have never realised what illness means to the breadwinner or the head of a household, who has neglected the happy assurance of recovery or the definite promise of cure that will come with time, but which never comes when indefinitely hoped for and solemnly deferred. We underestimate the personal equation, and it is the most serious defect in a physician, who can no more analyse the excellent results of sunshine of manner and sympathy than he can estimate the results of the sunshine of a spring morning to the invalid who has been long depressed by the gloom and depression of winter. To all this the pessimistic physician will object, "This is not scientific." It is not scientific in the sense that it can be made in a broth culture or dissected and demonstrated. But I venture to submit that our duty and satisfaction lie in curing our patients, and that we, by reducing everything to the material and palpable, to microscopes and cultures, if we do it to the neglect of other agencies of at least equally potent if less demonstrable effectiveness, are not only ineffectual for the cure of the sick, but, in just so much, unscientific. This vulgar insistence on the scientific, which excludes all that cannot be laid bare by the knife or the microscope, is a standing menace to the advance of medicine. The eager search for something that can be seen and handled, blinds us to mental and other influences, and possibly prevents the discovery of origins of disease and cures that might revolutionise medicine. Far be it from me to minimise the importance of the transcendent value of research, or to begrudge to the advance guard of science the merits of discoveries that have vastly helped the human race. What I protest against is that we should consider this all—the be-all and end-all—and the laboratory the only guide to the practice of clinical medicine. I have known some old worthies of medicine who without the "science," the pessimism, the world-weary despair, or the rather brutal indifference and lack of sympathy which occasionally characterise the man of the test-tube and the crucible, cured their patients, or permitted them to recover, if you will, and carried into the home that was devastated by disease a sense of security, of helpfulness and resource which were as well founded as, and are not excelled by, the most advanced physicians of to-day. They knew, too, how to handle drugs, and without the elegancies, the comprehensiveness, and the refinement of the modern pharmacy, worked miracles with either their drugs or their manner, I do not care which. To-day science is always being confounded at some corner by the quack and the charlatan inside and outside the profession, who makes a reputation on cures besides advertisements.

It is easy to laugh at them, to scoff at the empiric, to deride miraculous Lourdes or Holywell, but this sort of thing taps something in human nature that we do not always reach. It is perhaps hopeless to beg consideration for something that is not in fashion, and certainly it is not the fashion to think of anything but bacilli and micrococci, or to think of them in any other sense than the cause and origin, never the result and consequence of disease. But in spite of the unpopularity of the appeal, one may lift up a voice in the wilderness, and cry out for a little more hopefulness in the practice of medicine, a confidence which sometimes puts into the drug exhibited the potency for relief that may not be one of its inherent properties. If one could limit the pharmacopœia a little more, and handle a few drugs with the patience, observation, and interest devoted to the observation of laboratory experiments, there would be more chance of success.

The surgeon owes some of his success to the necessity he is under of considering his patient—him or herself—and having his germs, for the most part, in situations where he can deal with them or ignore them; see their effects uncomplicated by marvellously complex physical conditions by the great personal equation that baffles the physician. Being a surgeon, I have not these problems to face, and it is easy to be critical of those who must deal with them. But, under the infliction of the most grave surgical injuries, in the crises of disease that come under my observation, I cannot be blind to the immense influence of hope, of confidence on the part of the patient, of a serenity of mind that comes to those who are protected from outside disturbing influences. I see how a fit of temper or some mental worry will send up temperature, delay recovery, or even seriously endanger life itself; how a cheery nurse will save a case that despondency would kill; how the central organs of thought, of nervous control, and so forth, which we ignore while exclusively studying the life history and moral peculiarities of a germ, are ever exercising the most potent influence on the patient and reacting to the most imponderable and elusive stimuli in a pronounced and baffling manner. I hope that, having been surfeited with ideas, we are not running to the other extreme and insisting on a surfeit of killing, damning facts. Nothing could be more limiting, more crippling. The man of science who is capable of receiving ideas, founded, of course, on patient experiment and a sound basis of knowledge of the fundamentals of science, is the man who will succeed best with patients and achieve most for his art. Call me, if you will, a dreamer; say I am ignorant of the advance of preventive medicine, of the glorious possibilities before it, of germ theories and anti-toxins. Well, we do not cure consumption; we prevent it (which is better) and delay its progress. But we knew before any germ theories were heard of, or any bacillus tracked, that fresh air was practically the only thing that was of any use. We do not cure Bright's disease, or cancer, or heart disease. We discover no new drugs that have the efficacy of opium, quinine, arsenic, iron, and digitalis. But, engrossed in the search for some germ origin of many diseases, we are prone to forget the physician's function of friend, adviser, consoler, helper. In some respects, the physician should be like the good lawyer, whose client has been in trouble, but must be "got off," his burdens eased, his pains and penalties mitigated, his costs never taxed, his enemies confounded or befogged, his rights maintained against all comers. And if the ingenious lawyer cannot contrive it one way, he generally does it in another. We dwell too much on the little fleas that big fleas have on their backs to bite 'em, and ignore the lesser fleas *ad infinitum*, which, let us hope, tend to balance things. I want my physician, when it pleases God that I should suffer one, to try to look on me as a human being who wants helping over a stile and seeing through his trouble, germs and all. If he fold his arms and *say*, "Well, you know, we can only wait and see how things go," while he *looks*, "I wonder if we'll get a P.M.?" I shall want to die by myself, unblasted by the cruelty of scientific agnosticism and indifference.

I am quite certain, however, that this indifference is not due to any inhumanity. It is the result of the pessimistic attitude adopted by the newer school of physicians in too many instances. It is caused by the pathological bias, the concentration of the mind on lesions which do not appear to be easy subjects of attack by the armamentarium of even the new laboratories. We ignore the influence of the mind and the great nerve centres of the body, and should, I believe, by attacking disease on its more general aspects secure ultimate results that would surprise us by their success and their indifference to many germs which we suppose to be the absolute cause of disease in too many instances. At all events, I only venture, with much respect, to plead with my medical friends for a little more hopefulness in treatment, a more sanguine attitude with patients who are already depressed with disease and despair, and that reviving quality of confidence in oneself and one's profession that will do so much for patients whom our drugs cannot benefit.

